# The effect of target context and cue type in a postcue word pronunciation task

**DOI:** 10.2478/v10053-008-0086-0

**Published:** 2011-09-22

**Authors:** Karen Murphy, Lauren Green

**Affiliations:** 1 Applied Cognitive Neuroscience Research Unit, Behavioural Basis of Health, Griffith Health Institute; 2 School of Psychology, Gold Coast campus, Griffith University, Australia 4222

**Keywords:** postcue task, semantic priming, associate-semantic priming, word pronunciation, word recognition

## Abstract

Dallas and Merikle (1976a, 1976b) demonstrated that when participants were presented with a pair of words for over 1 s and subsequently cued to pronounce one of the words aloud (postcue task) semantic priming effects occurred. Humphreys, Lloyd-Jones, and Fias (1995) failed to replicate this postcue semantic priming effect using word pairs that were semantic category co-ordinates. The aim of Experiment 1 was to determine if the disparate postcue task results reported by these researchers could be accounted for by the prime-target contexts or cue types engaging different attentional processes or a combination of these factors. A postcue pronunciation task was used and word pairs presented were taken from an associate-semantic context and a semantic category context. In the Dallas and Merikle condition the line cue flanked the location in which the target word was previously shown. In the Humphreys et al. condition the cue word *UPPER* or *lower* was centrally presented and indicated the location in which the target word previously appeared. Results demonstrated that the occurrence of semantic and associate-semantic priming effects under postcue task conditions varied for the two cue types. Experiment 2 investigated if these results were attributable to a between subject manipulation of cue type. Using a fully repeated measures design priming effects were evident for top located targets in both the associate-semantic and semantic prime-target contexts. Experiment 3 used a between subjects design to rule out the possibility that carry over effects between cue and context conditions contributed to the postcue task priming effects. Priming was evident for top located targets in an associate-semantic and semantic context for the line cue. For the word cue there was priming for top located targets from an associate-semantic context and a reverse priming effect for top located targets from the semantic context. Possible explanations for the occurrence of priming effects under postcue task conditions are discussed.

## Introduction

*Semantic priming* refers to the finding that a target word (e.g., *bread*) is responded to more quickly after presentation of a related prime word (e.g., *butter*) than after presentation of an unrelated prime (e.g., *doctor*; Meyer & Schvaneveldt, 1971; see McNamara, 2005, and Neely, 1991, for reviews). Typically pronunciation or lexical decision tasks are used in studies of semantic priming; however postcue pronunciation tasks have been used to investigate prime-target context effects for word pronunciation (e.g., Balota, Boland, & Shields, 1989; Dallas & Merikle, 1976a, 1976b; Humphreys, Lloyd-Jones, & Fias, 1995). In a postcue task, participants are simultaneously presented with a pair of words for up to 1 s and subsequently cued to pronounce the target item indicated by the cue.

 The few studies that have examined context effects on word pronunciation in a postcue task have produced mixed results. For example, Dallas and Merikle (1976a, 1976b) demonstrated priming effects across four postcue task experiments. Balota et al. (1989) also reported a priming effect under postcue task conditions. In contrast, Humphreys et al. (1995; Experiment 4a) reported a 9 ms non-significant advantage when word targets were shown in a related compared to an unrelated context. The aim of this study was to examine if differences in cue type or prime-target contexts contributed to these disparate postcue task results.^1^

Different types of prime-target contexts have been used in semantic priming studies, including word pairs from the same semantic category (e.g., *fox-wolf*; semantically related primes and targets which are not associated), word pairs that are related by word association norms and not semantic category membership (e.g., *sweet-tooth*), and word pairs that share both a semantic category and word association context (e.g., *mum-dad*). Effects of associative relatedness have been demonstrated many times in the priming literature (for recent reviews see Lucas, 2000; McNamara, 2005). Additionally, some studies have demonstrated that for word pairs sharing a semantic and associative relationship there is a priming effect above that obtained for semantically related word pairs. This associative boost (Moss, Ostrin, Tyler, & Marslen-Wilson, 1995) has been confirmed in a meta-analysis (Lucas), which demonstrated priming effects twice the magnitude of those found in studies examining semantic priming. Early studies of semantic priming produced mixed results with some studies showing a priming effect (e.g., Fischler, 1977; McRae & Boisvert, 1998; Moss et al., Experiment 2) and other studies failing to do so (e.g., Lupker, 1984; Moss et al., Experiment 3; Shelton & Martin, 1992). Differences in methodologies and stimuli choice have contributed to the equivocal results for semantic priming (Lucas) and it has now been shown that semantic priming without association can be reliably produced with careful stimulus selection (see Lucas).

 Humphreys et al. (1995) suggested that the difference in results of their postcue task study compared to the experiments of Dallas and Merikle (1976a, 1976b) may have been due to the prime-target contexts. Humphreys et al. used word pairs that shared a categorical relationship (semantic context) while Dallas and Merikle used prime-target word pairs that were associatively and most likely semantically related (associate-semantic context). Thus it is possible that the larger significant priming effects reported by Dallas and Merikle were due to an associative boost beyond the semantic priming effect reported by Humphreys et al. It is also possible that associate-semantic but not semantic priming effects were evident in these studies due to activation of different word recognition stages. For example, Plaut (1995) has suggested that an associative context between items affects lexical access and a categorical context between items affects semantic memory. Thus perhaps only lexical access activation is evident under postcue task conditions. 

 Different cue types were also used in the Dallas and Merikle (1976a, 1976b) and Humphreys et al. (1995) studies. In the Dallas and Merikle experiments the cue was a horizontal line flanking the outside of the spatial location of the target word. As the cue and target appeared at the same spatial location participants should be able to process this type of cue automatically or involuntarily. This type of exogenous cue would capture attention at the target location and facilitate bottom-up processing (McCann, Folk, & Johnston, 1992; Henderson, 1991; Hauer & Macleod, 2006). In the Humphreys et al. experiment the cue was a centrally located word (*UPPER* or *lower*), which due to its symbolic nature would require some interpretation by the participant. Hence, attentional processes engaged by this endogenous cue type would be consciously controlled and as such driven by strategic goals (Fenske & Stolz, 2001; Henderson & Macquistan, 1993) and involve top-down processing. Thus, it is possible that the attentional processes involved in responding to the different cue types could contribute to the different results in the postcue tasks. 

The literature examining the effects of spatial attention on word recognition is mixed, with some studies indicating no role for spatial attention in word recognition (Allport, 1977; Sieroff & Posner, 1988) and others suggesting spatial attention is important for word recognition (Chiarello, Maxfield, Richards, & Kahan, 1995; McCann et al., 1992). Typically the studies investigating the effect of spatial cueing on word recognition have used precue tasks. That is, the cue was presented prior to the onset of the word(s) and the participant’s task was to identify the target word indicated by the precue, which was valid on some trials and invalid on others. While spatial attention may be important in precue word recognition tasks (e.g., McCann et al., 1992; Stolz & McCann, 2000; Stolz & Stevanovski, 2004), the effect of spatial attention on word recognition in a postcue task is unknown. Hence, to the authors’ knowledge this study will be the first to examine the impact of spatial attention and associate-semantic and semantic contexts on word pronunciation under postcue task conditions.

## Experiment 1

If the difference between the studies is due to cue type, there should be a significant priming effect across both prime-target contexts only in the Dallas and Merikle (1976a, 1976b) line cue condition, with the magnitude of this priming effect being greater for the associate-semantic condition than for the semantic condition due to an associative boost. However, if the difference is due to the prime-target context then there should be a greater priming effect in the associate-semantic than the semantic prime-target context in both cue tasks.

### Method

#### Participants

Sixty-four (47 female, 17 male) first year psychology students (*M* = 24.1 years of age, *SD* = 7.60) from Griffith University Gold Coast Campus voluntarily participated in this study in return for course credit. All participants had normal or corrected to normal vision and were native English speakers. The study was approved by the Human Ethics Research Committee Griffith University and all participants provided informed written consent prior to completing the study.

#### Stimuli

A total of 256 prime-target word pairs served as stimuli. All were common English words, ranging in length from three to eight letters.^2^

##### Semantic related words

 The 64 semantically related word pairs were taken from McRae, de Sa, and Seidenberg (1997) and from other word pairs that had been rated as having a high level of featural overlap in a pilot study. The prime-target word pairs in this condition were semantic category coordinates, shared numerous features and were not associated in either a forward or backward direction according to word association norms (Edinburgh Associative Thesaurus [EAT]; Computing and Information Systems Department [CISD], 1996). 

##### Associate-semantic related words

The 64 associate-semantic word pairs were semantic category coordinates that were also related by word association norms, with an association index of at least 50% (EAT; CISD, 1996). That is given the first word in each pair at least 50% of participants produced the second word in each pair in a word association task.

##### Semantic and associate-semantic unrelated words

Separately for each word list above, words within each pair were reassigned to target and prime words from another word pair. This created two sets of 64 word pairs that were not semantically or associatively related in either the forward or backward direction (EAT; CISD, 1996).

#### Stimulus presentation

To avoid repetition priming, each participant was presented with a target item once. Within each prime-target context, half the target word pairs were assigned to the related condition and the other half of the target items to the unrelated condition. This item condition assignment was then reversed for half of the participants, so that equally often a target appeared in the related and unrelated conditions. Across all conditions target items (semantic and associate-semantic) allocated to the related and unrelated conditions were matched on word frequency, letter number, and neighbourhood size (cf. Baayen, Piepenbrock, & VanRijn, 1993), and rotated across all possible stimulus presentation conditions to ensure that experimental version did not interact with any priming effects.^3^

**Figure 1.**
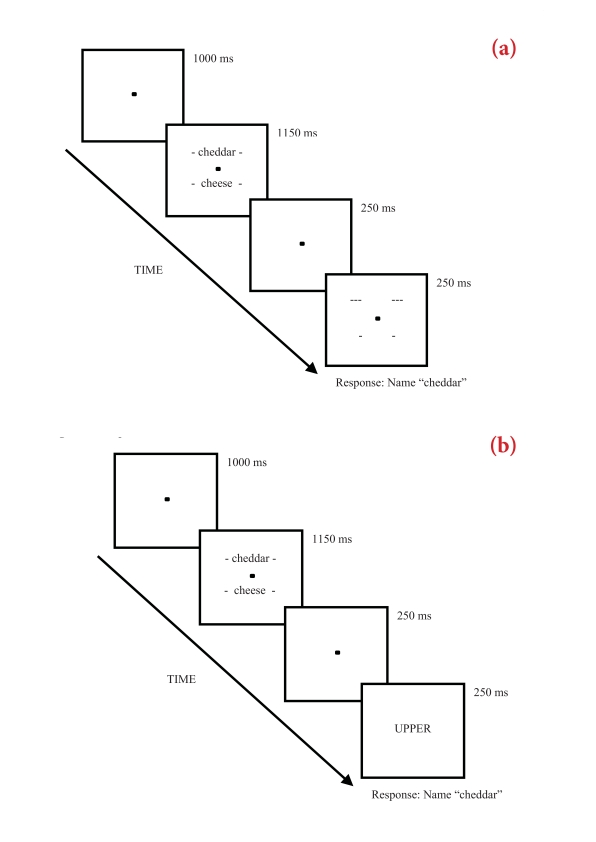
1a: Example of an associate-semantic related trial in the line cue condition where the target is the top located word *cheddar*. 1b: Example of an associate-semantic related trial in the word cue condition where the target is the top located word *cheddar*.

 Participants were seated 60 cm away from the computer screen so that each word subtended between 0.35° to 2.24° wide and 0.35° to 0.40° high of visual angle. The words were separated by 0.40° visual angle and the central fixation dot was located half way between the two words. Each word in the display was flanked either side by a single horizontal line (0.3° visual angle), to avoid lateral masking of the target item due to the presentation of the visual naming cue in the Dallas and Merikle (1976a, 1976b) line cue condition. In the line cue condition, the target cue subtended 0.95° visual angle each side of the target word. It was accompanied by a single small line either side of where the non-target had been located (spacing between lines was consistent for both items and based on the word with the greater number of letters), thus the target word was indicated by the location of the longer lines on the screen. In the Humphreys et al. (1995) condition, the word cue (centrally located display of *UPPER* or *lower*) subtended 0.4° of visual angle high and 2.0° of visual angle wide. Figure 1 shows the trial structure for both cue conditions. 

#### Apparatus and procedure

Stimuli were presented on a Hewlett Packard Ultra VGA 1024 computer monitor in lowercase white font on a black background, using the DMDX display system (Forster & Forster, 2003) run by a Hewlett Packard Intel Pentium II computer.

Each trial commenced with a centrally presented fixation point for 1000 ms. This was followed by the word pair and fixation point shown for 1150 ms, prior to a 250 ms display of only the fixation point. In the line cue condition, the fixation point remained on the screen, as the cue (lines) was presented for 250 ms, indicating participants should pronounce either the top or bottom word previously shown in the stimulus display. In the word cue condition, the word *UPPER* or *lower* was centrally presented for 250 ms. Once the cue disappeared from the screen a blank screen was presented until the next trial. The inter-trial interval was approximately 1 s and no feedback was presented after the trials. Word pronunciation latencies were recorded from the onset of the cue and the experimenter manually recorded the correctness of each response.

Participants were tested individually in a 30 min session. Thirty-two participants were randomly assigned to each cue condition. Except for differences in the type of cue used in the postcue tasks participants in the line cue and the word cue conditions completed identical versions of the experiment.

Prior to commencing the task participants were informed that they should silently read the two words once they had been displayed and then pronounce the target word as soon as possible once the cue was presented. Participants completed a set of 20 practice trials to familiarise themselves with the postcue task. Practice trials consisted of 10 related and 10 unrelated word pairs, with an equal number of semantic and associate-semantic related word pairs. None of the stimuli presented in the practice trials were presented in the experimental trials.

Each participant completed 138 experimental trials, consisting of two blocks of 69 trials. The first five trials in each block were filler trials. Participants were not informed that these were filler trials that were not included in the analysis. Within each block of trials, 16 word pairs were from the semantically related list, 16 were from the semantically unrelated list, 16 were from the associate-semantic related list, and 16 were from the associate-semantic unrelated list. Eight different versions of the experiment were created to ensure that each word pair was rotated across target locations (top and bottom) and so that each word served as the reported target word for half of the participants and the unreported prime word for the remaining participants. Equal numbers of participants were randomly assigned to each of the eight versions.

#### Design

The experiment used a mixed factorial design. There were three within subject factors: prime-target context (semantic or associate-semantic), relatedness (related or unrelated word pairs), and target location (top or bottom row of stimulus display). Cue type (line or word cue) was a between-subjects factor. The dependent variables were errors (percentage) and pronunciation latencies (milliseconds) for correct trials.

### Results

Only pronunciation latencies for correct target responses were included in the data set. Latencies less than 200 ms were removed from the data and remaining outlying latencies were replaced with latency values +/ 2.0 standard deviations from the mean for each individual participant. Only 2.97% of the latency scores were replaced in the line cue group, and 2.66% were replaced in the word cue group.

Minimal errors were evident for both cue types (line cue condition: 0.61% and word cue condition: 1.44% of total number of trials). Due to the extremely low number of errors no statistical analysis was conducted on these data. The pronunciation latency data were ana lysed using a 2 (cue type) x 2 (prime-target context) x 2 (relatedness) x 2 (target location) mixed factorial ANOVA.

The effect of cue type was significant, *F*(1, 62) = 42.17, *p* < .0005, η_p^2^_ = .41. The interaction between target location and cue type, *F*(1, 62) = 6.83, *p* = .01, η_p^2^_ = .10, revealed that although latencies were always shorter for the line cue condition compared to the word cue condition this effect was more evident for bottom located targets (*ps* < .05).

Pronunciation latencies were shorter for targets shown in the related than unrelated conditions, *F*(1, 62) = 45.02, *p* < .0005, η_p^2^_ = .42. The interaction between prime-target context and cue type, *F*(1, 62) = 4.86, *p* = .030, η_p^2^_ = .07, and the three-way interaction between relatedness, prime-target context, and cue type, *F*(1, 62) = 4.22, *p* = 0.044, η_p^2^_ = .06, were significant. To further examine this three-way interaction, semantic and associate-semantic priming effects were investigated for each cue type. For the line cue condition, there was priming for both prime-target contexts, with this effect being larger for the semantic than the associate-semantic word pairs. In the word cue condition, the priming effect was larger for the associate-semantic than the semantic prime-target context (all *ps* < .05). Refer to Table 1) for the relevant descriptive statistics.

**Table 1.** Mean Pronunciation Latency Data for the Relatedness by Cue Type by Prime-Target Context Interaction in Experiment 1.

	Line cue		Word cue	
	Related	Unrelated	Related	Unrelated
Prime-target context	Mean	Mean	Mean	Mean
Semantic	651 (14.61)	678 (14.78)	780 (14.61)	795 (14.78)
Associate-semantic	647 (14.28)	663 (13.91)	774 (14.28)	803 (13.91)

*Note*. Pronunciation latencies in milliseconds. Standard error in parentheses.

**Figure 2.**
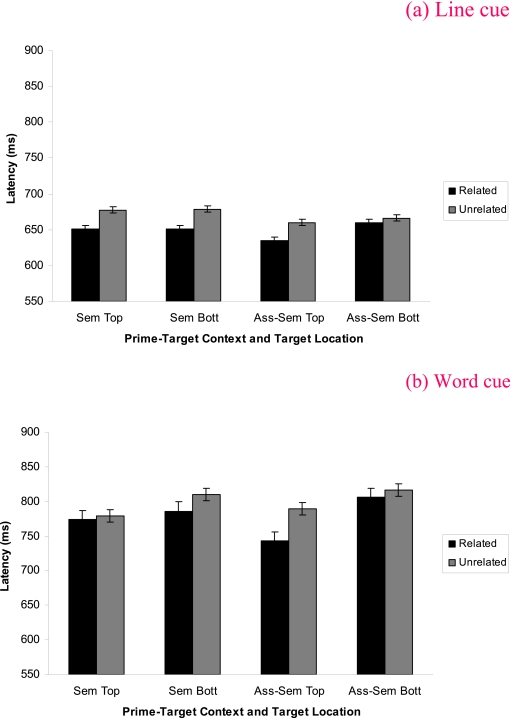
Experiment 1 mean (*SE*) word pronunciation latencies for the prime-target context by cue type by relatedness by target location interaction. Figure 2a shows the data for the line cue condition and Figure 2b shows the data for the word cue condition.

There was an effect of target location, *F*(1, 62) = 18.02, *p* < .0005, η_p^2^_ = .23, and prime-target context interacted with target location, *F*(1, 62) = 10.00, *p* = .002, η_p^2^_ = .14. The three-way interaction between prime-target context, target location, and relatedness was significant, *F*(1, 62) = 6.96, *p* = .011, η_p^2^_ = .10. There was a priming effect for both top and bottom located targets in the semantic condition although the priming effect was larger for the bottom located targets (*ps* < .05). In comparison for the associate-semantic items, there was only a significant priming effect for top located targets (*p* < .05). Refer to Table 2) for the relevant descriptive statistics. The four-way interaction was not significant, *F*(1, 62) < 1.5. Refer to Figure 2 for the relevant descriptive statistics for the four way interaction.

**Table 2.** Mean Pronunciation Latency Data for the Relatedness by Cue Type by Prime-Target Context Interaction in Experiment 1.

	Top located targets		Bottom located targets	
	Related	Unrelated	Related	Unrelated
Prime-target context	Mean	Mean	Mean	Mean
Semantic	713 (10.82)	728 (11.83)	718 (10.82)	744 (10.76)
Associate-semantic	689 (11.20)	725 (11.83)	733 (9.87)	741 (9.83)

*Note*. Pronunciation latencies in milliseconds. Standard error in parentheses.

### Discussion

The results showed that pronunciation latencies were always shorter for the line cue condition compared to the word cue condition. This outcome is consistent with the supposition that it would take longer to decode the word cue which would involve more controlled processing (e.g., Fenske & Stolz, 2001; Henderson & Macquistan, 1993), than the line cue which could be more automatically decoded (e.g., Hauer & MacLeod, 2006; Henderson, 1991; McCann et al., 1992).

There was a priming effect for both top and bottom located targets in the semantic condition although the priming effect was larger for the bottom located targets. In comparison for the associate-semantic items, there was only a priming effect for top located targets. These priming effects cannot be due to direction of association as there was no direction of association for the semantic word pairs and the direction of association for the stimulus displays was counterbalanced across participants in the associate-semantic condition. In general, these results suggest that word recognition processing benefits from a related distractor word and this effect is more evident for top located target words, perhaps suggesting some type of serial processing advantage for the first word processed in the display.

 Overall pronunciation latencies were shorter for targets shown in the related than the unrelated conditions, however the magnitude of the priming effects differed by prime-target context and cue type and were contrary to those hypothesized. For example, in the line cue condition, there was a larger priming effect for the semantic than the associate-semantic words pairs. In the word cue condition, there was a larger priming effect for associate-semantic than semantic items. These results suggest that semantic priming is larger with a cue that can be automatically processed. Hence one reason Humphreys et al. (1995) did not find significant semantic priming effects could have been due to the use of semantically related word pairs in a postcue task with a cue requiring decoding prior to response. Had they used a line cue as in the Dallas and Merikle (1976a, 1976b) experiments they may have reported significant semantic priming effects under postcue task conditions. Moreover, if Dallas and Merikle had used semantically related items in their postcue task, their priming effects may have been even larger than previously reported. It would therefore seem that priming effects under postcue task conditions are affected both by word context and cue type. Experiment 2 sought to further examine the impact of prime-target context and cue types on word pronunciation under postcue task conditions. 

## Experiment 2

 While Experiment 1 showed that priming effects under postcue task conditions were affected both by prime-target context and cue type, there is another possible explanation for these results. The Dallas and Merikle (1976a, 1976b) experiments only examined priming effects for associate-semantic word pairs and Humphreys et al. (1995) only investigated semantic priming effects under postcue task conditions. Thus in comparing the two studies both cue type and prime-target context were manipulated as between subject factors. Thus it is possible that individual differences across participant groups led to the dif- 

ferent priming results for the two studies. Experiment 2 manipulated all independent variables as within subjects factors in order to control for the impact of individual differences on priming effects under postcue task conditions.

### Method

#### Participants and stimulus presentation

Thirty-two (22 female, 10 male) first year psychology students (*M* = 22.06 years of age, *SD* = 6.70) from Griffith University Gold Coast Campus voluntarily participated in this study in return for course credit. All other participant characteristics were as for Experiment 1.

The same stimuli and cue conditions were used as in Experiment 1. In this experiment, participants were presented with each word twice, once in the line cue condition and once in the word cue condition. Participants only pronounced each word once to avoid repetition priming effects.

#### Procedure and design

Experiment 2 followed the same procedural information as Experiment 1 except where noted here. Participants were tested individually in a 1 hr session. Each participant completed both the line and word cue conditions and the order in which they completed each cue condition was counterbalanced across participants to minimise the impact of task practice effects on the data. Each participant completed two sets of 138 experimental trials, consisting of two blocks of 69 experimental trials.

The experiment used a fully repeated measures design. The within subject factors were: prime-target context (semantic or associate-semantic), relatedness (related or unrelated word pairs), target location (top or bottom row of stimulus display), and cue type (line or word cue). The dependent variables were errors (percentage) and pronunciation latencies (milliseconds) for correct trials.

### Results

Only pronunciation latencies for correct target responses were included in the data set. Latencies less than 200 ms were removed from the data and remaining outlying latencies were replaced with latency values +/ 2.0 standard deviations from the mean for each individual participant. This resulted in the replacement of 4.27% of the latencies in the line cue task, and 4.01% in the word cue task.

Minimal errors were evident across both cue types (line cue condition: 1.83% and word cue condition: 2.32% of total number of trials). Due to the extremely low number of errors no statistical analysis was conducted on these data. The pronunciation latency data were analysed using a 2 (cue type) x 2 (prime-target context) x 2 (relatedness) x 2 (target location) repeated measures ANOVA.

**Figure 3.**
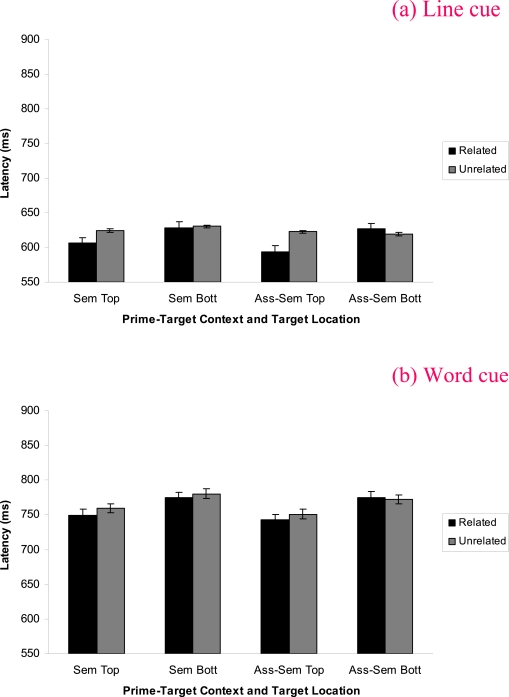
Experiment 2 mean (SE) word pronunciation latencies for the prime-target context by cue type by relatedness by target location interaction. Figure 3a shows the data for the line cue condition and Figure 3b shows the data for the word cue condition.

Pronunciation latencies were shorter in the line cue (*M* = 619, *SE* = 14.89) than the word cue condition (*M* = 763, *SE* = 17.33), *F*(1, 31) = 141.50, *p* < .0005, η_p^2^_ = .82; and there was an effect of the prime-target context, *F*(1, 31) = 4.19, *p* = .049, η_p^2^_ = .12, with shorter latencies being evident for words from the associate-semantic (*M* = 688, *SE* = 15.38) than semantic condition (*M* = 694, *SE* = 14.73). There was a main effect of target location, *F*(1, 31) = 9.92, *p* =.004, η_p^2^_ = .24, and pronunciation latencies were shorter for targets shown in the related than unrelated conditions, *F*(1, 31) = 4.87, *p* =.035, η_p^2^_ = .14. Target location and relatedness interacted, *F*(1, 31) = 7.85, *p* = .009, η_p^2^_ = .20, revealing a priming effect for top located targets (related: *M* = 673, *SE* = 15.15, vs. unrelated: *M* = 690, *SE* = 15.62) but not for bottom located targets (related: *M* = 701, *SE* = 15.51, vs. unrelated: *M* = 701, *SE* = 15.62). No other significant effects were found. Refer to Figure 3 for the relevant descriptive statistics for the four way interaction, *F*(1, 31) < 1.8.

### Discussion

Experiment 2 revealed that as for Experiment 1 pronunciation latencies were shorter for the line cue than word cue condition providing further support for greater task difficulty in decoding the word cue prior to pronouncing the target word aloud. In contrast to the first experiment, pronunciation latencies were shorter for words from the associate-semantic stimulus set compared to those from the semantic stimulus set regardless of a related or unrelated context. As the two sets of words were matched on factors that would have affected pronunciation latencies (e.g., number of letters, word frequency, and neighbourhood size) it is unclear why this difference occurred in the current experiment.

Experiment 2 sought to examine the possibility that the priming effects for the different cue types reported in the first experiment could have been due to individual differences. This experiment showed that priming effects occurred for both prime-target contexts for both the line and word cue but only for targets located in the top row of the stimulus display. Thus it is possible that the different prime-target priming effects observed within Experiment 1 were a function of individual differences between participants in the line and word cue tasks. Given that participants completed trials within all prime-target contexts and cue types in this experiment it is possible that the results of Experiment 2 could be due to an interaction effect of these factors producing priming effects. This idea will be tested in Experiment 3.

## Experiment 3

 Experiment 1 showed a larger priming effect for semantic than associate-semantic words in the line cue condition and greater priming for the associate-semantic than semantic conditions in the word cue task. Experiment 2 found priming effects for both contexts but only for top located targets. It is possible that the use of between and within subjects manipulations of cue type led to these different results. For example, given that the studies conducted by Dallas and Merikle (1976a, 1976b) and Humphreys et al. (1995) only used one cue type and one word type for each set of experiments it is possible that the results for the previous two experiments may be due to carry over or interactive effects when manipulating cue type and or word type as within subjects factors. Experiment 3 sought to provide a more direct comparison of the Dallas and Merikle and Humphreys et al. studies by manipulating both cue type and word type as between subjects factors.^4^

### Method

#### Participants and stimulus presentation

Sixty-four (52 female, 12 male) first year psychology students (*M* = 24.48 years of age, *SD* = 8.86) from Griffith University Gold Coast Campus voluntarily participated in this study in return for course credit. All other participant characteristics were as for Experiment 1.

The same stimuli and cue conditions were used as in Experiment 1. In this experiment participants only completed the task for one prime-target context and were presented with each word once to avoid repetition priming effects.

#### Procedure and design

Experiment 3 followed the same procedure as Experiment 1 except where noted here. Participants were tested individually in one 30 min session. Each participant completed the postcue task in one of the cue conditions (either line or word cue) and one the of prime-target contexts (either words from the semantic or associate-semantic set). Each participant completed one set of 69 experimental trials presented in a single block of trials. The first five trials of the block were filler items.

The experiment used a between subjects design for the prime-target context and cue type variables. Target location (top or bottom row of stimulus display) and relatedness (related or unrelated word pairs) were within subjects factors. The dependent variables were errors (percentage) and pronunciation latencies (milliseconds) for correct trials.

### Results

Pronunciation latencies less than 200 ms were removed from the data and remaining outlying latencies were replaced with latency values +/ 2.0 standard deviations from the mean for each individual participant. This resulted in replacement of 3.80% of the latency scores in the word cue associate-semantic context group, 4.59% in the line cue associate-semantic context group, 4.68% in the semantic word cue group, and 4.39% in the semantic line cue group.

Minimal errors were evident across conditions (line cue semantic: 1.56%, line cue associate-semantic: 1.66%, word cue semantic: 1.56%, and word cue associate-semantic: 1.95% of total number of trials per condition). Due to the extremely low number of errors no statistical analysis was conducted on these data. The pronunciation latency data were analysed using a 2 (cue type) x 2 (prime-target context) x 2 (relatedness) x 2 (target location) mixed factorial ANOVA.

The main effect of target location (top or bottom row of stimulus display) was significant, *F*(1, 60) = 27.98, *p* < .0005, η_p^2^_ = .32, as was the main effect of cue type, *F*(1, 60) = 56.51, *p* < .0005, η_p^2^_ = .49. The interaction between target location and cue type, *F*(1, 60) = 16.72, *p* < .0005, η_p^2^_ = .22, revealed no difference in pronunciation latencies for top and bottom located targets in the line cue task (p > .05). However, for the word cue task longer pronunciation latencies were evident for bottom compared to top located targets (*p* < .0005).

**Figure 4.**
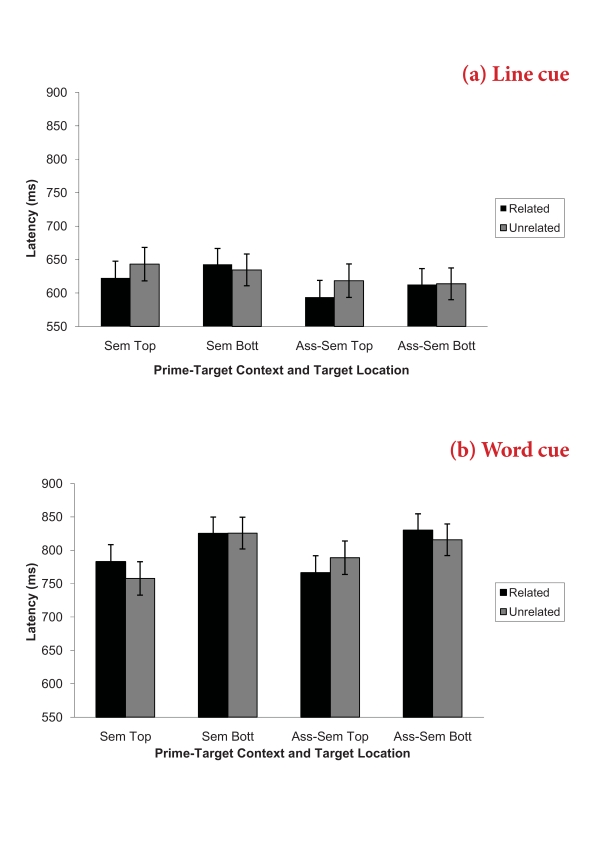
Experiment 3 mean (SE) pronunciation latencies for the prime-target context by cue type by relatedness by target location interaction. Figure 4a shows the data for the line cue condition and Figure 4b the data for the word cue condition.

The interaction between cue type and relatedness showed a marginal trend towards significance, *F*(1, 60) = 2.95, *p* = .091, η_p^2^_ = .05. The interaction between target location and relatedness approached significance, *F*(1, 60) = 3.50, *p* = .066, η_p^2^_ = .06, and revealed a significant priming effect for top located targets (related: *M* = 691, *SE* = 12.73; vs. unrelated: *M* = 702, *SE* = 12.53, *p* = .051) and no priming for bottom located targets (related: *M* = 728, *SE* = 12.32; vs. unrelated: *M* = 722, *SE* = 11.89, *p* = .399). The four way interaction between target location, cue type, word type, and relatedness was significant, *F*(1, 60) = 3.97, *p* = .051, η_p^2^_ = .06 (refer to Figure 4). Pairwise comparisons revealed that there was a priming effect in the line cue task for top located targets for both word types (associate-semantic: *p* = .025, semantic: *p* = .059). In the word cue task, there was a priming effect for the associate-semantic words for top located targets (*p* = .04) and for top located targets from the semantic items there was a reverse priming effect (*p* = .023).

### Discussion

As for the previous experiments the pronunciation latency data supported the notion of greater cue decoding requirements in the word than line cue tasks. This is further supported by the longer pronunciation latencies obtained for bottom compared to top located targets in the word cue task, where task difficulty would have been maximal. There was a priming effect for top located targets as in Experiment 2, however this depended on cue type and prime-target word context. Priming was evident for top located targets from the associate-semantic and semantic context in the line cue task. In the word cue task, while there was priming for top located targets from an associate-semantic context, there was a reverse priming effect for top located targets from the semantic context.

 While the priming effect for the associate-semantic condition is consistent with that reported by Dallas and Merikle (1976a, 1976b), it was only evident for top located targets. In addition the reverse priming effect for the word cue semantic prime-target context is not consistent with Humphreys et al. (1995) who showed a 9 ms non-significant priming effect. These results provide a partial replication of those reported by Dallas and Merikle and Humphreys et al., suggesting that some of the findings for Experiment 1 and 2 could be due to within subject manipulation of prime-target context and/or cue type. An overview of the results of all three experiments will be provided within the General Discussion. 

## General Discussion

The results of Experiment 1 demonstrated priming within the line cue condition for both prime-target contexts, with this effect being larger for the semantic than the associate-semantic words pairs. In the word cue condition, the priming effect was larger for associate-semantic than semantic word pairs. A larger priming effect was evident for bottom compared to top located targets within the semantic prime-target context, and only top located targets produced a significant priming effect for the associate-semantic items. In Experiment 2, for both the word and line cue tasks there was priming for top located targets for both the associate-semantic and semantic prime-target words. Experiment 3 produced priming for top located targets from an associate-semantic and semantic context for the line cue task, and in the word cue task there was priming for top located targets in the associate-semantic context. The word cue task revealed a reverse priming effect for top located targets from the semantic context.

 Although all experiments produced some differences in results, these could be accounted for by different factors being manipulated between and within subjects. Across all three experiments priming effects were consistently reported for top located targets. The use of a between subjects design for the cue and word type factors in Experiment 3 would have provided the most direct comparison with the results of Dallas and Merikle (1976a, 1976b) and Humphreys et al. (1995) . Although only evident for top located targets both prime-target contexts produced priming in the line cue task, replicating the associate-semantic priming effects reported by Dallas and Merikle. In addition priming was also evident for the word cue task for the associate-semantic items suggesting that Humphreys et al. may have reported priming if their word pairs had shared both an associative and semantic relationship. The reverse priming effect for the semantic items within the word cue task was not expected and the direction of this effect is not consistent with the 9 ms non-significant priming effect reported by Humphreys et al. Given this reverse priming effect was only evident in one experiment, replication of this result is required to ensure the reliability of this unexpected effect. 

 The occurrence of semantic or associative-semantic priming in postcue word pronunciation tasks cannot be explained by conventional accounts of semantic priming. For example, given that stimuli were displayed for over 1 s prior to cue onset it is unlikely that lexical access (which takes less than 200 ms; see Balota & Chumbley, 1985) accounts of semantic priming can explain these data (see McNamara, 2005, and Neely, 1991, for reviews). Humphreys et al. (1995) concluded that an associative context may exert an influence on word recognition processes such as orthography or the name retrieval stage in a postcue task, and that a semantic context between items did not affect the production of phonology in a postcue task. This proposal would explain the occurrence of the associate-semantic priming effect for the line and word cue task but it does not fully account for the occurrence of semantic priming effects only in the line cue task or the observation of these priming effects only for top located targets. 

Given that participants would have been encoding the words from the display in top to bottom order, the occurrence of priming for top located targets suggests that when accessing the target phonology there needs to be a related distractor following the target for priming to occur. For bottom located targets the need to process the irrelevant non-target top word before accessing the target phonology would appear to eliminate any priming. The occurrence of robust associate-semantic priming effects for both exogenous and endogenous cue types indicates that these priming effects are not affected by the attentional processes required to decode the cue and therefore do appear to be the result of access to the target’s phonology. In contrast, semantic priming effects appear to occur only when the cue can be automatically decoded which is consistent with the occurrence of automatic semantic priming effects within the word recognition literature (e.g., Fischler, 1977; Lucas, 2000; McRae & Boisvert, 1998; Moss et al., 1995, Experiment 2). The reverse priming effect obtained for the semantic items for top located targets suggests that when top down processes are required to decode the cue this results in an interference effect in accessing target phonology in a related compared to unrelated context. In other words, there is competition within the phonological output buffer, which may result through more controlled processing of the prime-target words. However it is unclear why this would only occur for top located targets unless as mentioned above any related context effect is lost by having to access the relevant target phonology in serial order.

The results from these experiments suggest that for associate-semantic items presented in a related context facilitation may result from access to the phonological lexicon or from residual activation from the semantic system to the phonological lexicon (e.g., Besner & Smith, 1992). In contrast, for semantic prime-targets the use of bottom up processes during cue decoding produces priming, whereas top down processes result in interference effects. Further research is required to determine the exact locus of these priming effects under postcue task conditions and in particular the occurrence of priming effects only for top located targets within the stimulus display.

## Footnotes

1 Stimulus display duration was another factor that differed between the studies of Dallas and Merikle (1976a, 1976b; 125/150 and 1150 ms) and Humphreys et al. (1995; 500 ms). However, it is unlikely that this experimental parameter is responsible for the equivocal postcue task priming effects reported in these studies as both short and long stimulus display durations have been shown to produce postcue task priming effects (Dallas & Merikle, 1976a, 1976b; Murphy, 2000).

2 Both Humphreys et al. (1995) and Dallas and Merikle (1976a) used words and non-words as stimuli. In order to simplify the design and to overcome some of the issues associated with the use of non-word stimuli reported by Dallas and Merikle (1976a) only word stimuli have been used have been used in this experiment.

3 Due to the use of asymmetrical associates, direction of association of word pairs was counterbalanced across subjects. Half the subjects were presented with the word pair displayed in the forward (downward) direction of association, the other half were presented with the word pair in the backward (upward) direction.

4 We thank an anonymous reviewer for suggesting a fully between subjects manipulation of cue and word type within this experiment.

## Author Note

The authors wish to thank Markus Kiefer and two anonymous reviewers for their helpful comments on earlier versions of this manuscript. Thanks also to Zoe Ward for her assistance with the data collection in Experiment 3.
